# Nævus

**Published:** 1848-01

**Authors:** 


*Nœvus.*—Some very singular examples of nævi materni are occasionally met with, but the most curious case of which we recollect to have ever heard, is described in a letter recently received by us from C. C. Porter, dentist, of Jacksonville, Ala. He says, "I operated on a lady's teeth about three months before her confinement, and among other operations, separated the right inferior bicuspides which were decayed on their sides carrying the file a little below the termination of the periosteum upon the necks of these teeth, giving her considerable pain, and causing her to become faint." On the birth of the child, an incision an eighth of an inch deep, from which blood was issuing, says, Mr. C. "precisely in the place in the gums of the inferior maxillary where the teeth of the mother had been separated." With regard to the cause of this singular phenomenon which we can regard in no other light than as a variety of nævus maturnus, it is, as in the case of other nævi, involved in too much obscurity for us to attempt to explain. Our correspondent will, therefore, please excuse us for not attempting to answer his "query," "the why and the wherefore of this singular occurrence?"

Nævi are supposed by some, to be produced by the operation of the mind or imagination of the mother upon the fœtus in utero, but this opinion is scouted by most medical writers. There are many circumstances, however, which go very far to favor this hypothesis.

